# Supplementary material for: uAUG creating variants in the 5’UTR of ENG causing Hereditary Hemorrhagic Telangiectasia
Source: NPJ Genom Med. 2023 Oct 17;8:32. doi: 10.1038/s41525-023-00378-5 (PMC10582052; doi:10.1038/s41525-023-00378-5)
Supplement: Supplementary file 1 — Supplementary Material [file 41525_2023_378_MOESM1_ESM.pdf]

uAUG creating variants in the 5'UTR of ENG causing Hereditary Hemorrhagic Telangiectasia

Omar Soukarieh[1,2*], Emmanuelle Tillet[3], Carole Proust[1], Charlène Dupont[1], Béatrice Jaspard-Vinassa[2], Florent Soubrier[4,5], Aurélie Goyenvalle[6], Mélanie Eyries[4,5], David-Alexandre Trégouët[1]

[1] Univ. Bordeaux, Inserm, Bordeaux Population Health Research Center, UMR 1219, F-33000 Bordeaux, France

[2] Univ. Bordeaux, INSERM, Biology of Cardiovascular Diseases, U1034, F-33600 Pessac, France

[3] INSERM UMR U1292, laboratoire BIOSANTE, Université Grenoble Alpes, Inserm, CEA, Grenoble, France

[4] Département de génétique, Hôpital Pitié-Salpêtrière, Sorbonne Université, Assistance Publique-Hôpitaux de Paris, Paris, France

[5] Sorbonne Université, INSERM, UMR_S 1166, Institute of Cardiometabolism And Nutrition (ICAN), Paris, France

[6] Université Paris-Saclay, UVSQ, Inserm, END-ICAP, Versailles, France

[*] corresponding author: omar.soukarieh@inserm.fr

Abstract

Hereditary Hemorrhagic Telangiectasia (HHT) is a rare, autosomal dominant, vascular disorder. About 80% of cases are caused by pathogenic variants in ACVRL1 (also known as ALK1) and ENG, with the remaining cases being unexplained. We identified two variants, c.-79C>T and c.-68G>A, in the 5'UTR of ENG in two unrelated patients. They create upstream AUGs at the origin of Overlapping upstream Open Reading Frames (uoORFs) ending at the same stop codon.

To assess the pathogenicity of these variants, we performed functional assays based on the expression of wild-type and mutant constructs in human cells and evaluated their effect on ALK1 activity in a BMP-response element assay. This assay is mandatory for molecular diagnosis and has been so far only applied to coding ENG variants.

These variants were associated with a decrease of protein levels in HeLa and HUVEC cells and a decreased ability to activate ALK1. We applied the same experiments on three additional uoORF-creating variants (c.-142A>T, c.-127C>T and c.-10C>T) located in the 5'UTR of ENG and previously reported in HHT patients. We found that all the analyzed variants alter protein levels and function. Additional experiments relying on an artificial deletion in our mutated constructs show that identified uAUGs could initiate the translation indicating that the associated effect is translation-dependent.

Overall, we have identified two 5'UTR ENG variations in HHT patients and shed new light on the role of upstream ORFs on ENG regulation. Our findings contribute to the amelioration of molecular diagnosis in HHT.

__Supplemental Figures and Tables__

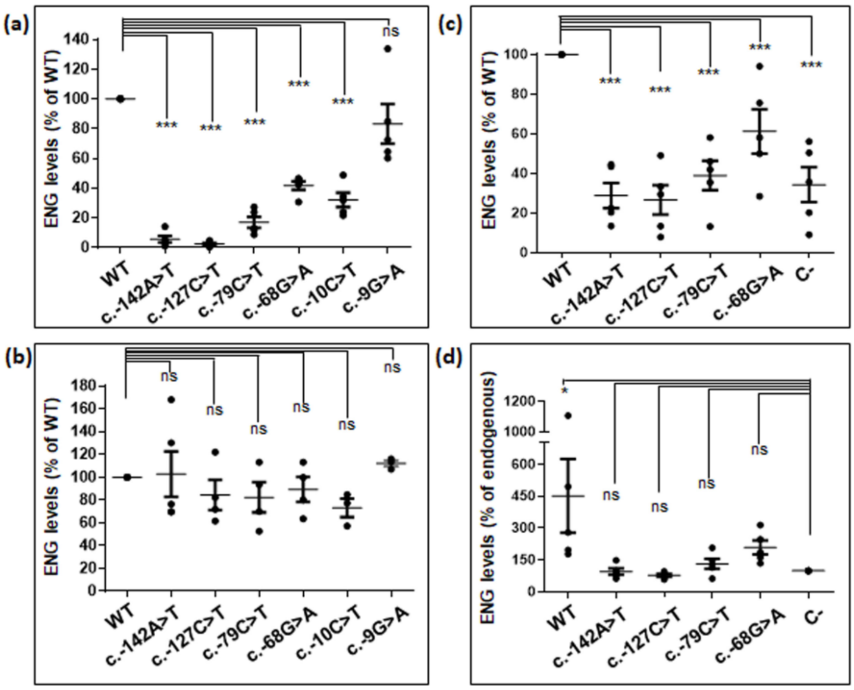

Supplemental figure 1. Quantification of ENG levels in HeLa (a-b) and HUVEC cells (c-d). (a) Apart for c.-9G>A variant, ENG steady-state levels in HeLa cells is significantly decreased with variants in comparison to the wild-type (WT) construct. For quantification, the average of each duplicate has been calculated from the quantified values and ENG levels for each sample have been normalized to the corresponding β-actin levels then to the WT (%).The two bands obtained for the Endoglin, corresponding to the more glycosylated (upper band) and less/non glycosylated (lower band) ENG monomers [1], were taken together for the quantification. (b) RNA levels of Endoglin do not variate between wild-type and variants. Normalized 2-ΔΔCT to the WT are shown. (c) Decrease of ENG protein levels in HUVECs cells in comparison to the wild-type (WT). ENG levels for each sample have been normalized to the corresponding β-actin levels then to the WT (%). C-, Negative control corresponding to the empty vector. (d) ENG levels associated to variants are similar or slightly higher of endogenous endoglin (C- on the Figure). ENG levels for each sample have been normalized to the corresponding β-actin levels then to the negative control (C-) (%). Graphs with standard error of the mean are representative of 5 independent experiments. ***, p-value < 10^{-3}, *, p < 5.10^{-2}, ns, non-significant (two-factor ANOVA followed by Tukey's multiple comparison test of variants versus WT (a, b, c) or negative control (d)).

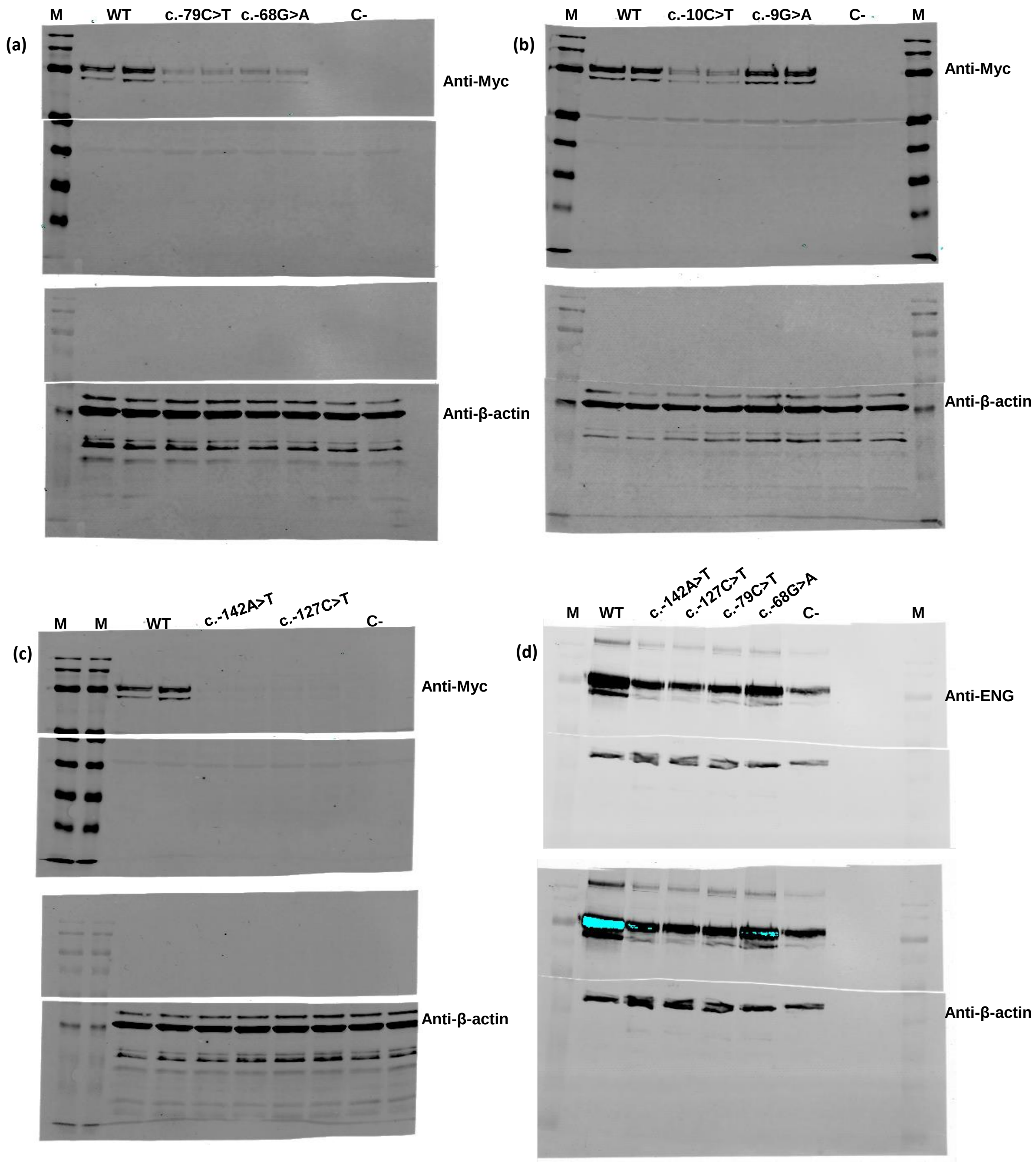

Supplemental figure 2. Western blot results on total proteins extracted from transfected HeLa cells with pcDNA3.1-L-ENG constructs (a, b and c) or from transduced HUVEC cells with lentiviruses containing ENG (d). The blots shown in a, b and c correspond to the uncropped upper, middle and lower blots in main Figure 1c, respectively and the blot shown in d corresponds to the uncropped blot in Figure 1d. Each blot was cut in 2 parts and each part was incubated with the corresponding antibody: monoclonal anti-(c-Myc Tag) (HeLa extracts), anti-ENG (HUVECs extracts) or anti-β-actin (HeLa and HUVECs extracts) as a loading control. Specific fluorescent secondary antibodies were used. Finally, Odyssey Infrared Imaging System (Li-Cor Biosciences) in 700 and 750 channels was used to scan, reveal, and quantify the blots. HeLa cells extracts were analyzed in duplicates. For HeLa cells, variants were analyzed in duplicates. M, protein ladder; WT, wild-type, C-, negative control corresponding to pcDNA3.1- empty vector.

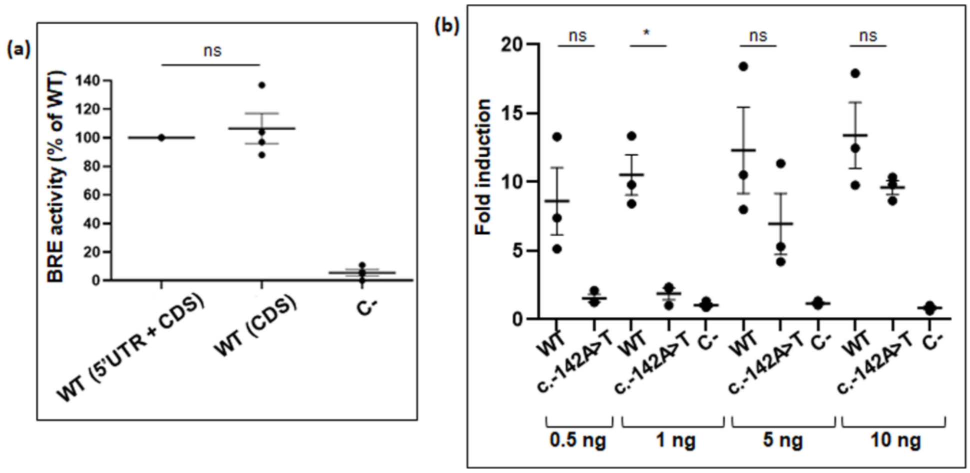

Supplemental figure 3. Optimization of cellular-based BRE activity assay to be applicable on *ENG* 5'UTR variants. (a) pcDNA3.1-L-ENG-WT vector gives similar BRE activity to ENG-WT plasmid only containing the CDS of ENG (WT(CDS)) used in Mallet et al., 2015 [1]. C- corresponds to pcDNA3.1 empty vector used as negative control. WT, wild-type; 5'UTR, 5' UnTranslated Region; CDS, CoDing Sequence. The graph with standard error of the mean is representative of 4 independent experiments. ns, non-significant (two-factor ANOVA followed by Tukey's multiple comparison test of WT(5'UTR+CDS) versus WT(CDS)). (b) Titration of ENG vectors with increasing quantities in order to determine optimal conditions to study 5'UTR variants. Fold induction corresponds to the detected activity normalized to control without ENG (C-). Transfected quantities of pcDNA3.1-L-ENG-WT or variant plasmids, and empty vector (C-) in 96 well plates are indicated. Graphs with standard error of the mean is representative of 3 independent experiments. ng, nanograms; ns, non-significant; *, $p < 5.10^{-2}$ (two-factor ANOVA followed by Tukey's multiple comparison test of c.-142A>T variant versus WT).

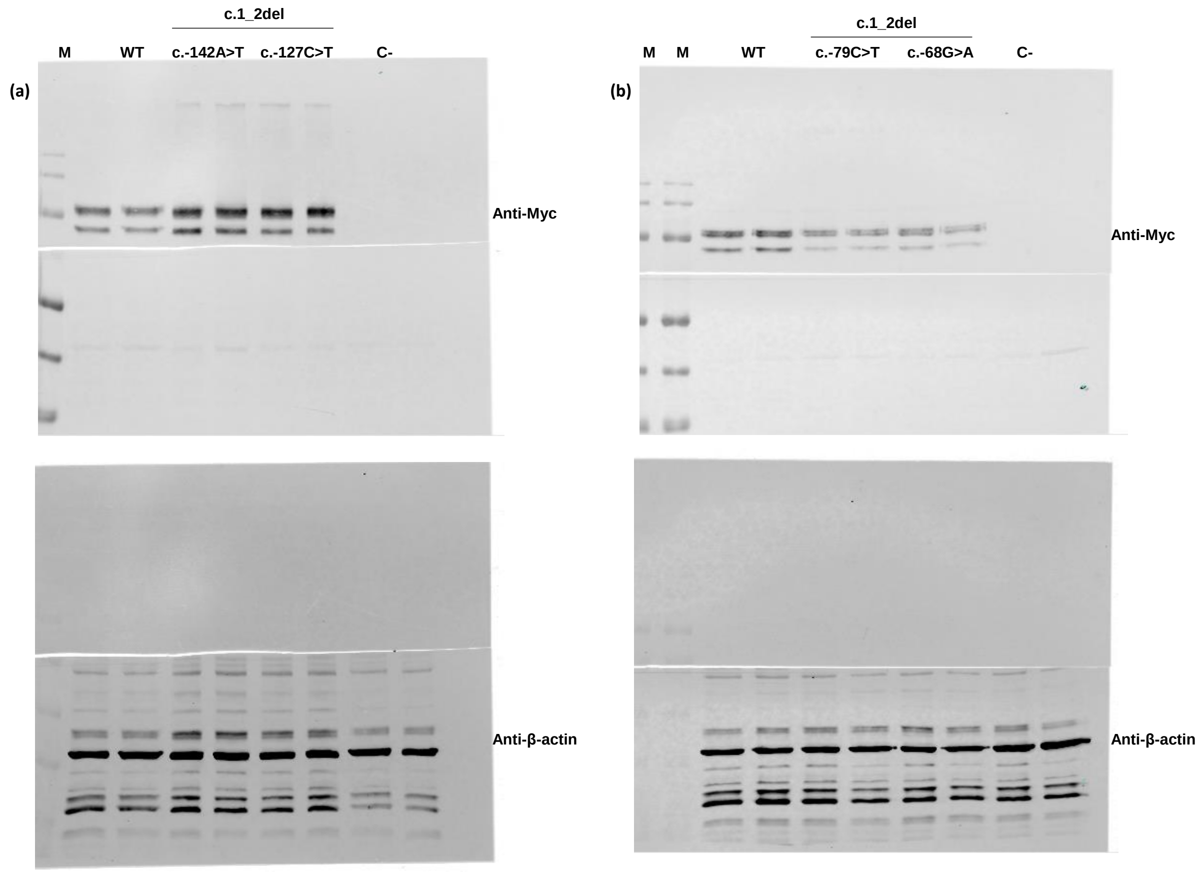

Supplemental figure 4. Western blot results on total proteins extracted from transfected HeLa cells with pcDNA3.1-L-ENG-c.1_2del constructs. The blots shown in a and b correspond to the uncropped right and left blots in main Figure 3b, respectively. Each blot was cut in 2 parts and each part was incubated with the corresponding antibody: monoclonal anti-(c-Myc Tag) or anti-β-actin as a loading control. Specific fluorescent secondary antibodies were used. Finally, Odyssey Infrared Imaging System (Li-Cor Biosciences) in 700 and 750 channels was used to scan, reveal, and quantify the blots. HeLa cells extracts were analyzed in duplicates. Each variant was analyzed in duplicate. M, protein ladder; WT, wild-type, C-, negative control corresponding to pcDNA3.1- empty vector.

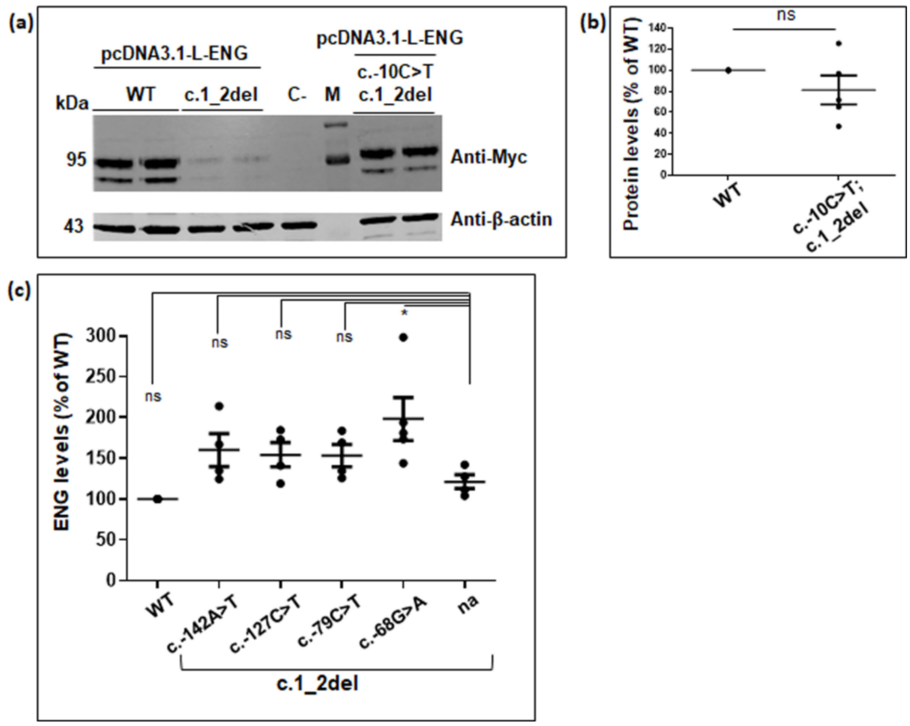

Supplemental figure 5. Created uAUGs in the 5'UTR of ENG seem to be able to initiate the translation. (a) Western blot results on total proteins extracted from transfected HeLa cells with 1 µg of pcDNA3.1-L-ENG constructs. Two bands of different molecular weights are observed for the Endoglin likely corresponding to more glycosylated (upper band) and less/non glycosylated (lower band) ENG monomers [1]. Anti-Myc and anti-βactin correspond to the used antibodies for the target and the reference proteins, respectively. kDa, kilodalton; M, protein ladder; WT, wild-type, C-, negative control corresponding to pcDNA3.1- empty vector. Uncropped blots are shown in Supp. figure 06. All blots were processed in parallel and derive from the same experiments. (b) Quantification of protein steady-state levels obtained in (a) and probably resulting from translation initiation at the created uAUG in presence of the c.-10C>T variant. For quantification, the average of each duplicate has been calculated from the quantified values and ENG levels have been normalized to the corresponding β-actin levels then to the WT (%). Graphs with standard error of the mean are representative of 5 independent experiments. *, p-value < 5.10^{-2}, ns, non-significant (two-factor ANOVA followed by Tukey's multiple comparison test of variants versus WT). (c) Quantification of ENG RNA levels in HeLa cells transfected with pcDNA3.1-L-ENG constructs containing the c.1_2del deletion in presence or in absence (na) of uAUG-creating variants. Normalized 2-ΔΔCT values to the WT are shown. Graphs with standard error of the mean are representative of 5 independent experiments. *, p-value < 5.10^{-2}, ns, non-significant (two-factor ANOVA followed by Tukey's multiple comparison test of variants versus na corresponding to the construct only containing the c.1_2del).

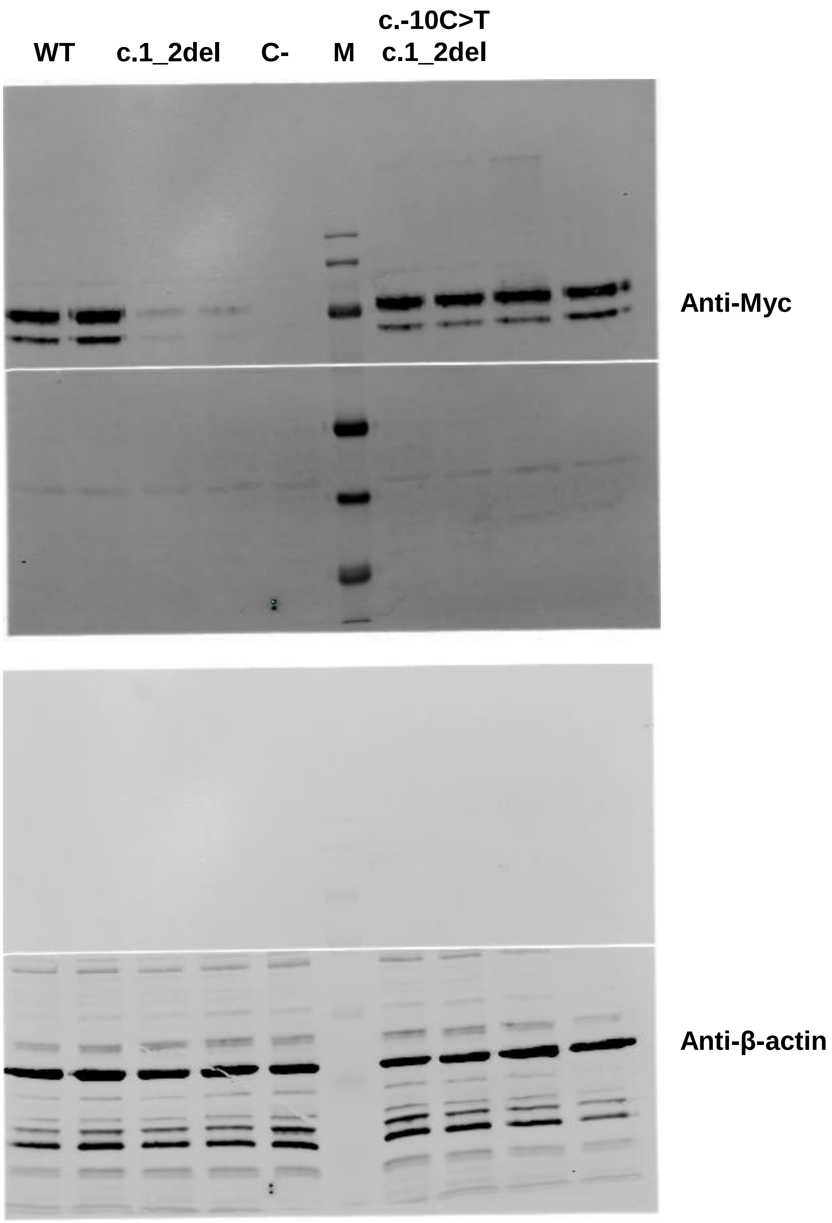

Supplemental figure 6. Western blot results on total proteins extracted from transfected HeLa cells with pcDNA3.1-L-ENG-c.1_2del constructs. The shown blot corresponds to the uncropped blot in Supp. figure 04a. The blot was cut in 2 parts and each part was incubated with the corresponding antibody: monoclonal anti-(c-Myc Tag) or anti-β-actin as a loading control. Specific fluorescent secondary antibodies were used. Finally, Odyssey Infrared Imaging System (Li-Cor Biosciences) in 700 and 750 channels was used to scan, reveal, and quantify the blots. HeLa cells extracts were analyzed in duplicates. Each variant was analyzed in duplicate. M, protein ladder; WT, wild-type, C-, negative control corresponding to pcDNA3.1- empty vector.

Supplemental Table 2. Bioinfomatics predictions and kozak sequences of the created uAUGs in ENG. PreTIS translation confidence scores for created uAUGs are indicated. Optimal Kozak sequence surrounding an AUG as defined in Kozak, 1984 [2] was taken into account c**R**ccAUG**g** (R = purine, A or G) to compare the sequences surrounding the created uAUGs. Nucleotides at positions -3 and +4 relative to the predicted uAUGs are in bold [3,4]. Position the uAUG-creating variant is underlined. The sequence surrounding the main AUG of ENG is c**a**gcATG**g.** *, variant position in the L-ENG; NM_001114753.3 transcript (c.1 corresponds to the A of the main AUG).

**Position***	**PreTis Score (Interpretation)**	**Kozak Sequence**
c.-142A>T	0.84 (High)	c**c**agAAG**g**
c.-127C>T	0.84 (High)	a**g**ggACG**c**
c.-79C>T	0.67 (Low)	g**c**ccACG**c**
c.-68G>A	0.89 (High)	c**c**cgGTG**c**
c.-10C>T	0.85 (High)	c**c**ccACG**t**
c.-9G>A	0.96 (High)	c**c**acGTG**g**

Supplemental Table 3. Gene content of the used custom Next Generation Sequencing panel. Specific HHT genes are bolded.

Gene name
ACVRL1
AQP1
ATP13A3
BMP10
BMPR2
CAV1
EIF2AK4
ENG
EPHB4
FOXF1
GDF2
GLMN
KCNK3
KDR
PTEN
RASA1
SMAD4
SMAD9
SOX17
TBX4
TEK

Supplemental Table 4. Primers used in this study for cloning, qPCR, back-to-back directed mutagenesis (DM) and sequencing. Restriction sites are underlined. Variations are in lowercase. Position of c.1_2del is indicated with a dash.

Technique	Plasmid	Primer name	Primer sequence (5'-3')
Cloning	pcDNA3.1 & MND14	ENG-Iso1-WT-BamHI-F	tagtggatccATACCACAGCCTTCATCTGCGC
Cloning	pcDNA3.1	ENG-Iso1-WT-HindIII-R	gcccaagcttTGCCATGCTGCTGGTGGAG
Cloning	pRRLsin-MND-MCS-WPRE	ENG-Iso1-NheI-R	gcaggctagcCTATGCCATGCTGCTGGTGGAG
Cloning	pGL3b	ENG-Prom-MluI-F	tcttacgcgtGGATCCCAGCGCTACCATCTTC
Cloning	pGL3b	ENG-Prom-XhoI-R	agatctcgagGCTGTCCACGTGGGGGC
qPCR	Human ENG	ENG-qPCR-Ex9-F	ACAAGTTTGTCTTGCGCAGT
qPCR	Human ENG	ENG-qPCR-Ex11-R	CTGTCCATGTTGAGGCAGTG
qPCR	Human α-Tubulin	hu_TUBA F	GATGCTGCCAATAACTATGCCCGAG
qPCR	Human α-Tubulin	hu_TUBA R	GAAAACCAAGAAGCCCTGAAGACGG
DM	pcDNA3.1-ENG	ENG_c.-142AT-F	CACCCCAGAtGGCTGGAGC
DM	pcDNA3.1-ENG	ENG_c.-142AT-pR	GCGGCCGAGGGGTCAG
DM	pcDNA3.1-ENG	ENG_c.-127CT-F	GAGCAGGGAtGCCGTCGC
DM	pcDNA3.1-ENG	ENG_127CT-pR	CAGCCTTCTGGGGTGGC
DM	pcDNA3.1-ENG	ENG-c.-79CT-F	GAGCCCAtGCCGGCC
DM	pcDNA3.1-ENG	ENG-c.-79CT-pR	GCACGGGGACCCGAGGGG
DM	pcDNA3.1-ENG	ENG-c.-68GA-F	GGCCCCGaTGCCCGC
DM	pcDNA3.1-ENG	ENG-c.-68GA-pR	GGCGTGGGCTCGCACGGG
DM	pcDNA3.1-ENG	ENG-c.-10CT-F	CAGGCCCCCAtGTGGACA
DM	pcDNA3.1-ENG	ENG-c.-9GA-F	CAGGCCCCCACaTGGACA
DM	pcDNA3.1-ENG	ENG-c.-10_-9-pR	TGCGCTGGGCCTTATCCTG
DM	pcDNA3.1-ENG	ENG-c.1_2delAT-F	CACGTGGACAGC-GGACCGCGGC
DM	pcDNA3.1-ENG	ENG-c.-2_-1delGC-pR	GGGGCCTGTGCGCTGG
Sequencing	Human ENG	ENG-seq1-F	CATGTCCTCTTCCTGGAGTTC
Sequencing	Human ENG	ENG-seq2-F	ATGTCCTTGATCCAGACAAAG
Sequencing	pcDNA3.1	CMV-F	CGCAAATGGGCGGTAGGCGTG
Sequencing	pcDNA3.1	BGH-R	TAGAAGGCACAGTCGAGG
Sequencing	pRRLsin-MND-MCS-WPRE	Fvf MND BclXl	ACCCTGTGCCTTATTTGAAC
Sequencing	pRRLsin-MND-MCS-WPRE	verifMNDhGli3	GGCATTAAAGCAGCGTATCC
Sequencing	pRRLsin-MND-MCS-WPRE	R Arhgef11 MND	ATCCACATAGCGTAAAAGGAGCA
Sequencing	pGL3b	GL primer2	CTTTATGTTTTTGGCGTCTTCCA
Sequencing	pGL3b	RV primer 3	CTAGCAAAATAGGCTGTCCC

Bibliography

1. Mallet, C. *et al.* Functional analysis of endoglin mutations from hereditary hemorrhagic telangiectasia type 1 patients reveals different mechanisms for endoglin loss of function. *Hum Mol Genet* **24**, 1142–1154 (2015).
2. Kozak, M. Compilation and analysis of sequences upstream from the translational start site in eukaryotic mRNAs. *Nucleic Acids Res* **12**, 857–872 (1984).
3. Kozak, M. Point mutations define a sequence flanking the AUG initiator codon that modulates translation by eukaryotic ribosomes. *Cell* **44**, 283–292 (1986).
4. Kozak, M. An analysis of 5'-noncoding sequences from 699 vertebrate messenger RNAs. *Nucleic Acids Res* **15**, 8125–8148 (1987).
