# Supplementary material for: uAUG creating variants in the 5’UTR of ENG causing Hereditary Hemorrhagic Telangiectasia
Source: NPJ Genom Med. 2023 Oct 17;8:32. doi: 10.1038/s41525-023-00378-5 (PMC10582052; doi:10.1038/s41525-023-00378-5)
Supplement: Supplementary file 2 — Reporting Summary [file 41525_2023_378_MOESM2_ESM.pdf]

nature portfolio

Corresponding author(s): Omar Soukarieh

Last updated by author(s): Jun 7, 2023

Reporting Summary

Nature Portfolio wishes to improve the reproducibility of the work that we publish. This form provides structure for consistency and transparency in reporting. For further information on Nature Portfolio policies, see our Editorial Policies and the Editorial Policy Checklist.

Statistics

For all statistical analyses, confirm that the following items are present in the figure legend, table legend, main text, or Methods section.

n/a	Confirmed	
☐	☒	The exact sample size (*n*) for each experimental group/condition, given as a discrete number and unit of measurement
☐	☒	A statement on whether measurements were taken from distinct samples or whether the same sample was measured repeatedly
☐	☒	The statistical test(s) used AND whether they are one- or two-sided<br>*Only common tests should be described solely by name; describe more complex techniques in the Methods section.*
☐	☒	A description of all covariates tested
☐	☒	A description of any assumptions or corrections, such as tests of normality and adjustment for multiple comparisons
☐	☒	A full description of the statistical parameters including central tendency (e.g. means) or other basic estimates (e.g. regression coefficient) AND variation (e.g. standard deviation) or associated estimates of uncertainty (e.g. confidence intervals)
☐	☒	For null hypothesis testing, the test statistic (e.g. *F*, *t*, *r*) with confidence intervals, effect sizes, degrees of freedom and *P* value noted<br>*Give P values as exact values whenever suitable.*
☒	☐	For Bayesian analysis, information on the choice of priors and Markov chain Monte Carlo settings
☒	☐	For hierarchical and complex designs, identification of the appropriate level for tests and full reporting of outcomes
☒	☐	Estimates of effect sizes (e.g. Cohen's *d*, Pearson's *r*), indicating how they were calculated

Our web collection on statistics for biologists contains articles on many of the points above.

Software and code

Policy information about availability of computer code

Data collection	No software was used.
Data analysis	No software was used.

For manuscripts utilizing custom algorithms or software that are central to the research but not yet described in published literature, software must be made available to editors and reviewers. We strongly encourage code deposition in a community repository (e.g. GitHub). See the Nature Portfolio guidelines for submitting code & software for further information.

Data

Policy information about availability of data

All manuscripts must include a data availability statement. This statement should provide the following information, where applicable:

- Accession codes, unique identifiers, or web links for publicly available datasets
- A description of any restrictions on data availability
- For clinical datasets or third party data, please ensure that the statement adheres to our policy

ENG constructs generated during this study are available upon request.

Research involving human participants, their data, or biological material

Policy information about studies with human participants or human data. See also policy information about sex, gender (identity/presentation), and sexual orientation and race, ethnicity and racism.

Reporting on sex and gender	ENG variants affecting we investigated in our study have been found both in male and female HHT patients. In sum, 70% of carriers i58% of published cases are females. All the experimental investigations hold for both genders.
Reporting on race, ethnicity, or other socially relevant groupings	No categorization variables have been used.
Population characteristics	In this study, 274 individuals with rare vascular diseases among which 53 are with suspected HHT have been screened for candidate pathogenic variants.<br>We identified two very rare heterozygous candidate variants located in the 5'UTR of ENG in two unrelated individuals with unresolved molecular diagnosis for HHT among the 274 sequenced individuals.<br>The carrier of ENG c.-79C>T was diagnosed with HHT as she presented with epistaxis, mucosal and cutaneous telangiectasias and a pulmonary arteriovenous malformation that leaded to stroke. Her 3 monozygotic triplet sons were examined and had epistaxis, and one of them had 2 cutaneous telangiectasias suggestive of HHT. The carrier of the c.-68G>A variant presented with several telangiectasias, a pulmonary arteriovenous malformation and a familial history of epistaxis since her paternal grand-father, her father, her brother and her daughter were reported to have recurrent epistaxis.
Recruitment	Recruited as part of a molecular diagnosis routine conducted at the genetics department of the Pitié-Salpêtrière Hospital (Paris, France).
Ethics oversight	The patients provided written informed consent for their DNA material to be used for genetic analysis in the context of molecular diagnosis in accordance with the French bioethics' laws (Commission Nationale de l'Informatique et des Libertés n° 1774128).

Note that full information on the approval of the study protocol must also be provided in the manuscript.

Field-specific reporting

Please select the one below that is the best fit for your research. If you are not sure, read the appropriate sections before making your selection.

☒ Life sciences ☐ Behavioural & social sciences ☐ Ecological, evolutionary & environmental sciences

For a reference copy of the document with all sections, see nature.com/documents/nr-reporting-summary-flat.pdf

Life sciences study design

All studies must disclose on these points even when the disclosure is negative.

Sample size	As part of a molecular diagnosis routine conducted at the genetics department of the Pitié-Salpêtrière Hospital (Paris, France), 274 individuals with rare vascular diseases among which 53 are with suspected HHT have been screened for candidate pathogenic variants using a custom next-generation sequencing (NGS) targeted gene panel including HHT genes (ACVRL1, ENG and SMAD4, GDF2, RASA1 and EPHB4, between others) and additional genes related to other hereditary vascular diseases. Sequencing was performed on genomic DNA extracted from whole blood. VCF files from sequenced individuals were scrutinized using the MORFEE bioinformatics tool 25 in order to detect and annotate non coding SNVs creating uAUGs (uAUG-SNVs) in the 5'UTR of the sequenced genes.
Data exclusions	No exclusion criteria.
Replication	All transfections and transductions have been performed in duplicate and all experiments have been repeated at least 3 times as indicated in the main text and in supplemental data.
Randomization	This is not relevant to our study. We performed in vitro functional assays and compared samples in the same conditions each time.
Blinding	This is not relevant to our study. We performed in vitro functional assays and compared samples in the same conditions each time.

Reporting for specific materials, systems and methods

We require information from authors about some types of materials, experimental systems and methods used in many studies. Here, indicate whether each material, system or method listed is relevant to your study. If you are not sure if a list item applies to your research, read the appropriate section before selecting a response.

Materials & experimental systems

n/a	Involved in the study
☐	☒ Antibodies
☐	☒ Eukaryotic cell lines
☒	☐ Palaeontology and archaeology
☒	☐ Animals and other organisms
☐	☒ Clinical data
☒	☐ Dual use research of concern
☒	☐ Plants

Methods

n/a	Involved in the study
☒	☐ ChIP-seq
☒	☐ Flow cytometry
☒	☐ MRI-based neuroimaging

Antibodies

Antibodies used	Mouse purified monoclonal anti-(c-Myc Tag) antibody (Merck Millipore, clone 9E10 | 05 419) (HeLa extracts).<br>Rabbit anti-ENG (Rabbit monoclonal [EPR10145-12] to CD105) (Abcam, ab169545, lot: GR3922132-3) (HUVEC extracts).<br>and anti-β-actin (b-Actin (13E5) Rabbit mAb, Cell Signaling) (HeLa and HUVECs extracts)
Validation	Anti-(c-Myc Tag) antibody is reactive in all species as reported at the manufacturer website.<br>Rabbit anti-ENG has been validated on HeLa and HUVECs extracts in Western blot by the manufacturer.<br>Anti-β-actin: validated on different human extracts in Western blot by the manufacturer.

Eukaryotic cell lines

Policy information about cell lines and Sex and Gender in Research

Cell line source(s)	HeLa, epithelial human cells isolated from a cervical carcinoma derived from a 31-year-old patient (ATCC).<br>Human Umbilical Vein Endothelial Cells (HUVEC) from pooled donors (Lonza).
Authentication	None of the cell lines used were authenticated.
Mycoplasma contamination	All cell lines were tested negative for mycoplasma contamination.
Commonly misidentified lines<br>(See ICLAC register)	None of the cell lines used were misidentified.

Clinical data

Policy information about clinical studies
All manuscripts should comply with the ICMJE guidelines for publication of clinical research and a completed CONSORT checklist must be included with all submissions.

Clinical trial registration	This is not a clinical trial.
Study protocol	As part of a molecular diagnosis routine conducted at the genetics department of the Pitié-Salpêtrière Hospital (Paris, France), 274 individuals with rare vascular diseases among which 53 are with suspected HHT have been screened for candidate pathogenic variants using a custom next-generation sequencing (NGS) targeted gene panel.<br>The patients provided written informed consent for their DNA material to be used for genetic analysis in the context of molecular diagnosis in accordance with the French bioethics' laws (Commission Nationale de l'Informatique et des Libertés n° 1774128).
Data collection	Clinical data were communicated by the genetics department of the Pitié-Salpêtrière Hospital (Paris, France).
Outcomes	Clinical diagnosis of HHT is determined based on the Curaçao criteria established by the HHT international committee.
